# Are form priming effects phonological or perceptual? Electrophysiological evidence from American Sign Language

**DOI:** 10.1016/j.cognition.2021.104979

**Published:** 2021-12-11

**Authors:** Gabriela Meade, Brittany Lee, Natasja Massa, Phillip J. Holcomb, Katherine J. Midgley, Karen Emmorey

**Affiliations:** a Joint Doctoral Program in Language and Communicative Disorders, San Diego State University & University of California, San Diego, United States of America; b Department of Psychology, San Diego State University, United States of America; c School of Speech, Language, and Hearing Sciences, San Diego State University, United States of America

**Keywords:** Phonological priming, Sign language, ERPs, N400

## Abstract

Form priming has been used to identify and demarcate the processes that underlie word and sign recognition. The facilitation that results from the prime and target being related in form is typically interpreted in terms of pre-activation of linguistic representations, with little to no consideration for the potential contributions of increased perceptual overlap between related pairs. Indeed, isolating the contribution of perceptual similarity is impossible in spoken languages; there are no listeners who can perceive speech but have not acquired a sound-based phonological system. Here, we compared the electrophysiological indices of form priming effects in American Sign Language between hearing non-signers (i.e., who had no visual-manual phonological system) and deaf signers. We reasoned that similarities in priming effects between groups would most likely be perceptual in nature, whereas priming effects that are specific to the signer group would reflect pre-activation of phonological representations. Behavior in the go/no-go repetition detection task was remarkably similar between groups. Priming in a pre-N400 window was also largely similar across groups, consistent with an early effect of perceptual similarity. However, priming effects diverged between groups during the subsequent N400 and post-N400 windows. Signers had more typical form priming effects and were especially attuned to handshape overlap, whereas non-signers did not exhibit an N400 component and were more sensitive to location overlap. We attribute this pattern to an interplay between perceptual similarity and phonological knowledge. Perceptual similarity contributes to early phonological priming effects, while phonological knowledge tunes sensitivity to linguistically relevant dimensions of perceptual similarity.

## 1. Introduction

A significant linguistic discovery was that all human languages, including sign languages, exhibit structure at the level of form. Research on sign languages over the last few decades has documented the existence of segmental structure (e.g., Liddell & Johnson, 1989; Sandler, 1986; Stokoe, 1960), syllabic structure (e.g., Brentari, 1998), moraic structure (e.g., Perlmutter, 1992), a sonority hierarchy (e.g., Brentari, 1993), and phonological constraints (e.g., Sandler & Lillo-Martin, 2006). The fact that phonological structure exists in soundless languages is a testament to the fundamental importance of this level of linguistic structure. However, much less is known about how this phonological structure affects the neuro-cognitive underpinnings of the perception and recognition of signs. Here, we used event-related potentials (ERPs) and a priming paradigm to compare the effects of form overlap on sign recognition in deaf signers, who had acquired the phonology of American Sign Language (ASL), and hearing non-signers, who had no knowledge of ASL phonology. The priming paradigm lends insight into how form overlap with a prime sign influences perception and processing of a target sign. Comparing these priming effects between signers and non-signers allowed us to differentiate between the perceptual and linguistic components of sign recognition, with the former being shared between groups and the latter being unique to the signers, who possess a phonological system in the visual-manual modality.

**Fig. 1.**
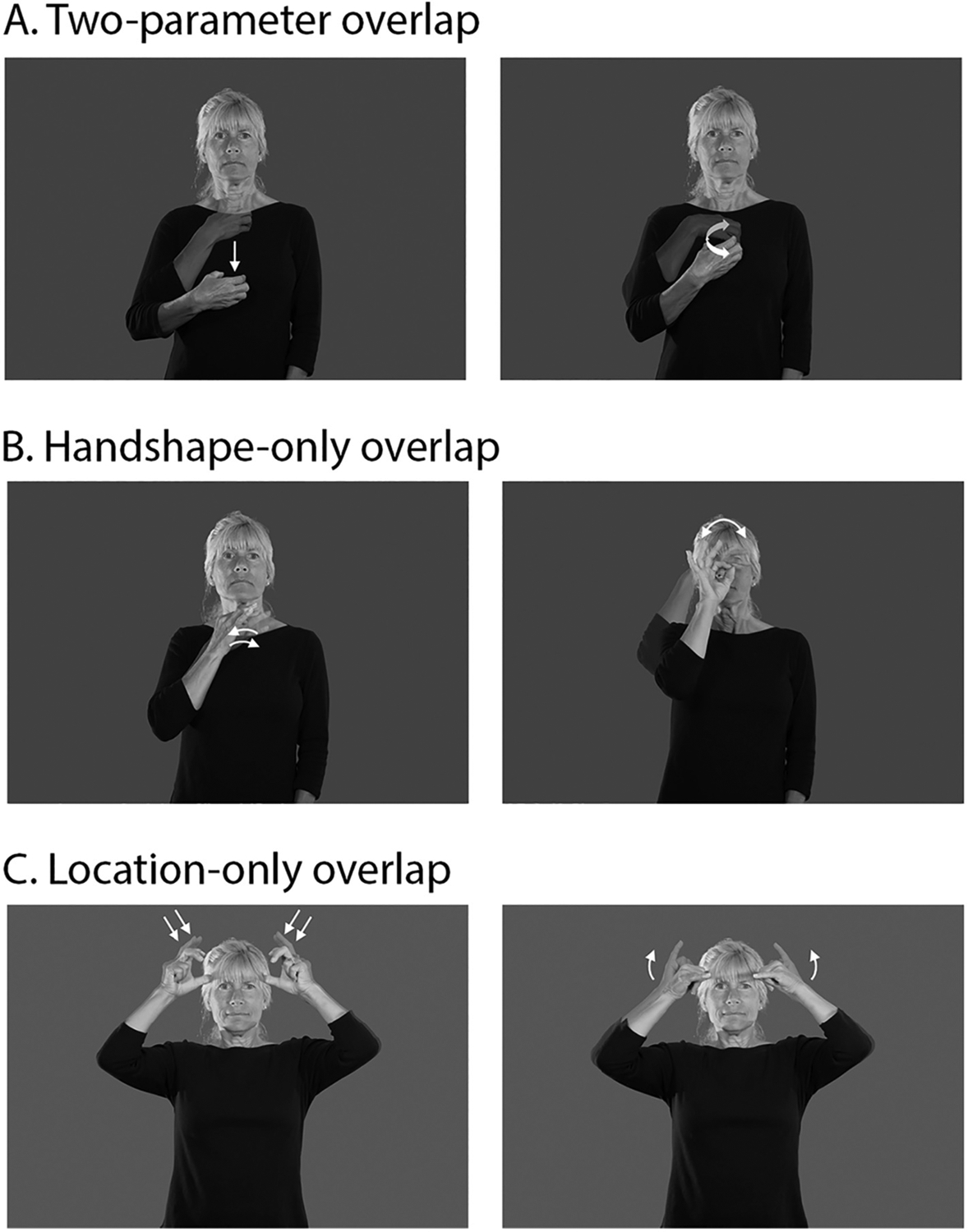
Example stimuli. The ASL signs HUNGRY and COUGH share both handshape and location, but differ in movement (A), whereas the ASL signs CURIOUS and FOX share only handshape (B) and the ASL signs DEVIL and COW share only location (C).

As a brief introduction to sign phonology, the segmental units of signs are often referred to as parameters. Signs are composed of a combination of three primary parameters: handshape, location, and movement (orientation is another parameter, but is often analyzed as a subfeature of handshape; Brentari, 1998). Signers, like speakers, must be able to rapidly identify and segment phonological form in order to access lexical representations (e.g., Orfanidou, Adam, Morgan, & McQueen, 2010) and to distinguish between minimal pairs that differ by only a single parameter. An example of a sign minimal pair in ASL is illustrated in Fig. 1A; the only parameter that differentiates the ASL signs for HUNGRY and COUGH is movement.

The phonological relationship between signs affects processing in ways that bear similarities with findings for spoken words. In recent studies, processing has generally been facilitated when target signs are preceded by prime signs that share two parameters compared to phonologically unrelated prime signs, as reflected in faster responses and smaller amplitude N400s (e.g., Baus, Gutiérrez, & Carreiras, 2014; Corina & Knapp, 2006; Dye & Shih, 2006; Gutiérrez, Müller, Baus, & Carreiras, 2012; Meade et al., 2021; Meade, Lee, Midgley, Holcomb, & Emmorey, 2018). The N400 is a peak in the ERP waveform that occurs approximately 400 ms after word or sign onset and is associated with lexico-semantic processing (see, e.g., Kutas & Federmeier, 2011). This component is temporally sandwiched between, and influenced by, earlier perceptual processing and later decision-making. These sign priming results are reminiscent of the well-established rhyme priming effect in spoken languages; spoken and written words preceded by a rhyming prime word also elicit smaller amplitude negativities compared to those preceded by phonologically unrelated prime words (e.g., Coch, Grossi, Coffey-Corina, Holcomb, & Neville, 2002; Grossi, Coch, Coffey-Corina, Holcomb, & Neville, 2001; MacSweeney, Goswami, & Neville, 2013; Perrin & García-Larrea, 2003; Weber-Fox, Spencer, Cuadrado, & Smith, 2003).

However, visual-manual parameters are not completely analogous to phonemes (see, e.g., Sandler & Lillo-Martin, 2006). One of the major distinctions is that signs are composed of multiple parameters that occur with some degree of simultaneity rather than in succession. Phonological priming effects in sign language differ depending on which of these parameters is manipulated (e.g., Baus et al., 2014; Baus, Gutiérrez-Sigut, Quer, & Carreiras, 2008; Corina & Emmorey, 1993; Corina & Knapp, 2006; Dye & Shih, 2006; Gutiérrez et al., 2012; Mayberry & Witcher, 2005; Meade et al., 2021). For example, growing evidence suggests that interference, rather than priming, occurs when only the location parameter is shared between prime and target signs (e.g., Carreiras, Gutiérrez-Sigut, Baquero, & Corina, 2008; Corina & Emmorey, 1993). In contrast, when only the handshape parameter overlaps, either no priming or facilitation is observed (e.g., Carreiras et al., 2008; Corina & Emmorey, 1993; Dye & Shih, 2006).

In addition, there is evidence to suggest that knowledge of a sign language affects the relative importance that is assigned to each parameter and how they are each processed. Location is acquired earlier and more accurately than handshape by adult second-language learners (Chen Pichler & Koulidobrova, 2015), suggesting that this phonological parameter may be more perceptually salient to non-signers. Non-signers also appear to be more sensitive to location than handshape overlap when making perceptual decisions about signs. Non-signers rate pseudosigns that share location as more similar than those that share handshape (Hildebrandt & Corina, 2002). In contrast, signers have been found to exhibit categorical perception effects for handshape, but not for location (Emmorey, McCullough, & Brentari, 2003), suggesting that signers are perceptually tuned to linguistically distinctive handshapes (see also Palmer, Fais, Golinkoff, & Werker, 2012). Signers have also been found to create more robust memory traces for visually presented hands compared to non-signers (Peressotti, Scaltritti, & Miozzo, 2018) and are better able to discriminate between anatomically possible and impossible hand gestures, showing early visual sensitivity to anatomical violations (Almeida, Poeppel, & Corina, 2016). Thus, linguistic knowledge may differentially affect processing of each parameter and, by extension, the respective priming effects.

Across signed and spoken modalities, phonological priming effects are typically explained in terms of sublexical and lexical representations. Comparing the rhyme priming effects elicited by words versus pseudowords has informed our understanding of the relative contributions at these two levels of processing. For example, Dumay et al. (2001) found that rhyme priming effects were significantly larger for words than for pseudowords. They argued that phonological priming is likely due to at least two separate mechanisms. One of the mechanisms is sublexical: “intermediate representations already activated or computed during prime processing would be more rapidly available for target identification” (p. 136). This mechanism contributes to phonological priming irrespective of lexicality. The second mechanism acts at the level of lexical selection and serves to increase the size of the priming effect for words relative to pseudowords. When Gutiérrez et al. (2012) extended this approach to sign language, they also found evidence of different priming patterns as a function of lexicality (see also Dye & Shih, 2006). In particular, the effect of location overlap related to lexical competition (i.e., increased N400 negativity) was observed for sign, but not pseudosign, targets. Together then, these results suggest that some aspect of the phonological priming effect occurs at the lexical level, but that other aspects of the effect occur for non-lexicalized items that conform to the phonological rules of a familiar language.

This interpretation in terms of linguistic representations disregards the fact that phonologically related pairs of signs or words also share more perceptual features than unrelated pairs. A major aim of the present study was to examine the extent to which non-linguistic visual similarity contributes to the phonological priming ERP effect in signed languages. To achieve this, we examined the effects of handshape and location priming in groups of signers and non-signers. Non-signers are unique in that they are able to visually perceive signs, but they have not acquired a visual-manual phonological system. Thus, any ERP priming similarities between non-signers and signers are likely due to perceptual processing, rather than phonological processing. Distinguishing between phonological and perceptual priming effects like this in spoken languages is virtually impossible as there is no control group that can perceive speech but has not acquired a sound-based phonological system.

### 1.1. Present study

Taken together, there are well-established ERP effects of phonological relatedness that are generally similar irrespective of language modality. Phonological priming effects with pseudowords and pseudosigns indicate that part of the effect originates at a pre-lexical level. However, existing studies have not been able to disentangle the effect of perceptual similarity from sublexical pre-activation because the pseudowords were always composed of sublexical units that were familiar to the participants. Here, we compared ERP phonological priming effects between hearing individuals who had no formal experience with ASL and proficient deaf ASL signers. Both groups performed a go/no-go repetition detection task that did not require knowledge of ASL or guessing on the part of non-signers.

To the extent that the N400 phonological priming effects observed in proficient signers (e.g., Gutiérrez et al., 2012; Meade et al., 2018; Meade et al., 2021) are actually driven by phonological overlap, they should only be observed in signers. In addition, an early (pre-N400) phonological priming effect might be shared by the two groups and reflect sensitivity to perceptual similarity. A candidate component for this effect might be the N300, which has previously been shown to be sensitive to form-level effects in sign language (e.g., Emmorey, Winsler, Midgley, Grainger, & Holcomb, 2020; Meade et al., 2018). In contrast, if perceptual similarity is not a major contributing factor to phonological priming or if early effects are associated with phonological overlap at the sublexical level, then phonological relatedness may not influence sign processing in the non-signer group in either the N300 or N400 time windows. Finally, we expected that phonological overlap would affect sign processing for both non-signers and signers in a later (post-N400) time window that is associated with decision-related processes. Given the extant literature suggesting that sensitivity to handshape and location is modulated by sign knowledge, we included both types of overlap in addition to a condition in which both handshape and location overlapped between prime and target.

## 2. Methods

### 2.1. Participants

Participants included 20 hearing non-signers (12 female; mean age 31.2 years; *SD* 5.4 years) who reported never being formally exposed to ASL and 20 severely-to-profoundly deaf signers (10 female; mean age 32.8 years; *SD* 7.1 years) who began learning ASL before the age of seven. Four deaf participants had deaf parents (and were exposed to ASL from birth) and 16 had hearing parents, with a mean age of ASL exposure of 2 years old (range: birth to 6 years old). Both groups participated in the same experimental protocol. Data from the deaf signers were previously reported by Meade et al. (2021) and are presented here for comparison with the hearing non-signer data. All of the hearing participants were right-handed, as were all but four of the deaf participants. Participants in both groups had normal or corrected-to-normal vision per self report and were volunteers who provided informed consent in accordance with the Institutional Review Board at San Diego State University. Data from an additional seven hearing participants were excluded from analyses due to high artifact rejection rates (> 25%; *N* = 2), falling asleep during the experiment (*N* = 1), or experimenter error (*N* = 4). Similarly, data from two deaf participants were excluded due to high artifact rejection rates.

### 2.2. Stimuli

Critical stimuli were the same as in the study reported by Meade et al. (2021) and are described in detail there. A total of 138 ASL sign triplets (e.g., HUNGRY, SEE, COUGH) were used to form two sign pairs with the same target (e.g., HUNGRY-COUGH, SEE-COUGH). One of the pairs in each triplet was phonologically related (e.g., HUNGRY-COUGH have the same handshape and location in ASL; see Fig. 1A), and the other was phonologically unrelated (e.g., SEE-COUGH do not overlap in any phonological parameters in ASL). Phonologically related sign pairs fell into one of three conditions with 46 targets per condition (see Fig. 1): handshape-only overlap (HS), location-only overlap (LOC), and handshape-location overlap (HS + LOC). None of the related primetarget pairs overlapped in movement, and none of the unrelated pairs overlapped in any of the three phonological parameters. An additional 46 repetition probe trials were included for the go/no-go repetition detection task but not analyzed. These pairs consisted of two different exemplars of the same sign (e.g., FLOWER-FLOWER). A list of the English glosses corresponding to Entry IDs for videos in the ASL-LEX database (asl-lex.org) or in Signbank (aslsignbank.haskins.yale.edu) can be found at https://osf.io/xchsy/. A native ASL signer was filmed producing each of the signs at a natural rate. Each video was clipped to begin two frames before sign onset and end at sign offset. Sign onsets and offsets were determined as in previous studies (see, e.g., Caselli, Sevcikova Sehyr, Cohen-Goldberg, & Emmorey, 2017; Meade et al., 2018).

### 2.3. Procedure

During the experiment, participants were seated in a comfortable chair in a dimly lit room. Participants saw pairs of sign videos and pressed a button on a videogame response controller if the two signs were the same. They were warned that some of the sign pairs might look similar due to overlap in handshape, movement, and/or location, and were instructed to only press the button if the signs were exactly the same. No response was required on the critical trials. This task was chosen because it does not require knowledge of ASL and could therefore be completed by both groups. Instructions were provided in written and oral English for the non-signers. They were given in written English and ASL for the signers with a deaf native signer present to answer any questions.

As in our previous ASL phonological priming studies (e.g., Meade et al., 2018; Meade et al., 2021), each trial consisted of a prime video and a target video that were separated by a 1300 ms stimulus onset asynchrony (SOA; i.e., a prime video followed by a blank screen of variable duration). A grey rectangle that subtended a visual angle of 10.8 degrees in the vertical direction and 14.0 degrees in the horizontal direction remained at the center of the black screen for the duration of each trial. The model subtended a visual angle of approximately 9.7 degrees in the vertical direction and 4.9 degrees in the horizontal direction within the grey rectangle. She was not visible in the time interval between signing the prime and target signs. A black screen appeared immediately after the target video and remained for 800 ms to minimize artifacts during the epoch of interest. In between trials, a purple fixation cross appeared for 1500 ms and a white fixation cross appeared for 500 ms followed by a blank screen for 500 ms. Participants were asked to blink during the purple fixation cross in between trials and during longer breaks that occurred approximately every 20 trials. Trials were arranged into two pseudorandomized lists; one list was the reverse order of the other. Each trial occurred once in each half of the experimental list (i.e., twice total for each participant). Per condition, 23 of the 46 targets in the first half of the experiment were presented with phonologically related primes and the remaining 23 were presented with unrelated primes. Whether any given target appeared first with a related prime or unrelated prime was counterbalanced across participants. We presented each target twice to ensure that all other possible confounding factors (e. g., visual complexity, frequency, concreteness, iconicity, etc.) were controlled in the analyses of interest. The experiment began with a practice list that had eight sign pairs, two of which were repetition probes and none of which occurred during the main experiment.

### 2.4. EEG recording and analysis

EEG was recorded from 29 active electrodes in an Electro-Cap. Additional electrodes were placed beside the outer canthus of the right eye (to monitor for horizontal eye movement), below the left eye (to monitor for blinks in conjunction with forehead electrodes), on the left mastoid (reference), and on the right mastoid (to measure for differential mastoid activity). Scalp and mastoid electrode impedances were maintained below 2.5 kΩ and eye electrodes below 5 kΩ. EEG was amplified by a SynAmpsRT amplifier (Neuroscan-Compumedics) with a bandpass of DC to 100 Hz and was continuously sampled at 500 Hz.

**Fig. 2.**
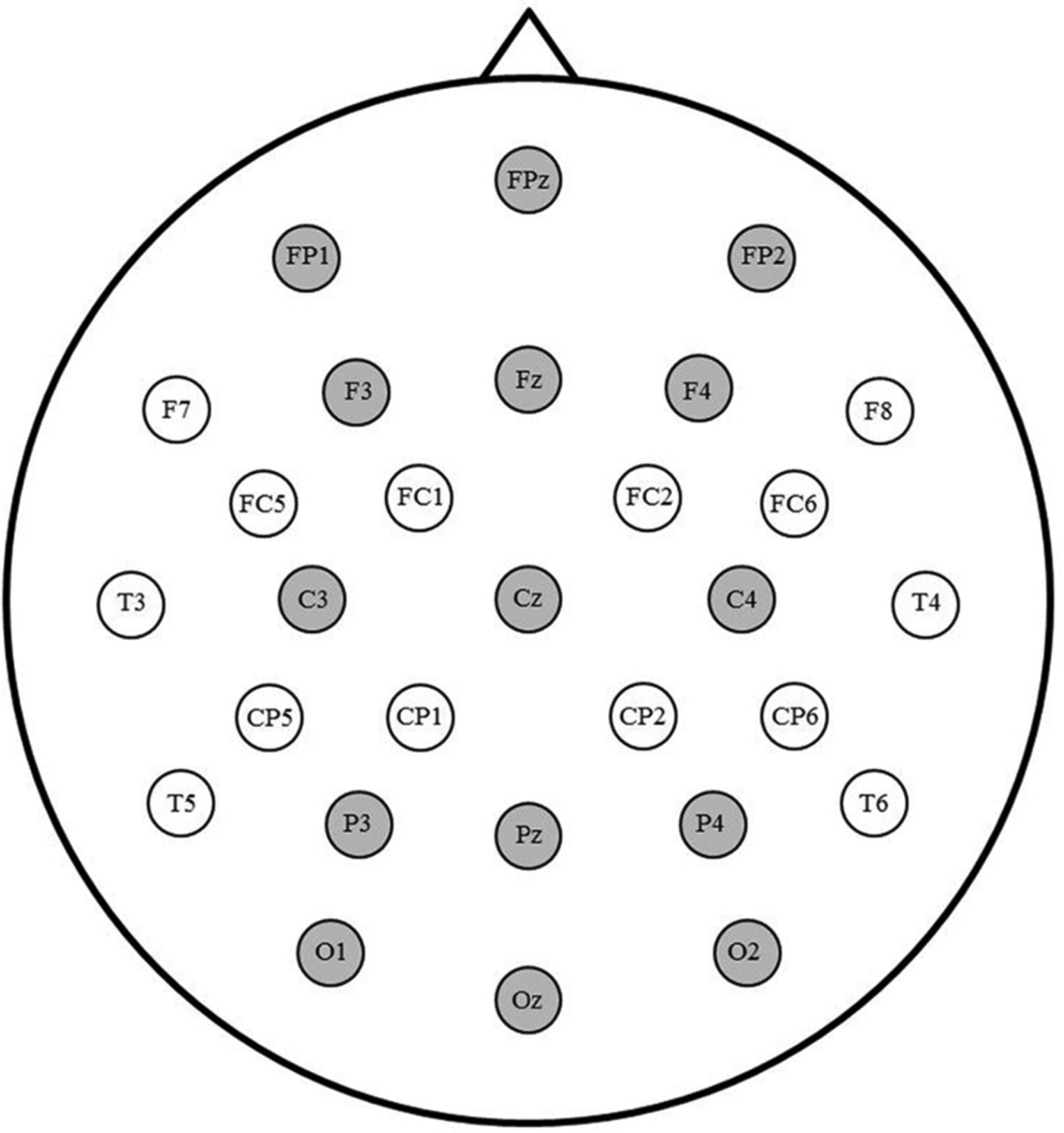
Electrode montage. The 15 sites included in analyses are highlighted in grey.

ERPs time-locked to target video onset and referenced to the left mastoid were averaged separately for each condition and processed with a 15 Hz low-pass filter. Trials contaminated by eye movement or drift artifact during the 100 ms pre-stimulus baseline or within 900 ms of target video onset were rejected prior to averaging. An average of 17 trials (6%) were rejected for artifacts in the non-signer group, and 26 trials (10%) were rejected in the signer group. Critical trials with button presses (i.e., false alarms) were also rejected. Repeated measures analyses of variance (ANOVAs) were used to analyze the data from 15 electrode sites illustrated in Fig. 2 (see also, e.g., Lee, Meade, Midgley, Holcomb, & Emmorey, 2019; Meade et al., 2021). Based on visual inspection of the grand average waveforms of the signer group, which were also presented by Meade et al. (2021), mean N400 amplitude was calculated between 400 and 600 ms. We also report mean amplitude in a pre-N400 window (200–400 ms) that encompasses the N300 (see also Emmorey et al., 2020; Meade et al., 2018) and might be more sensitive to early perceptual overlap between related signs as well as a post-N400 window (600–800 ms) that was expected to better reflect decisional processes. Individual data can be found at https://osf.io/xchsy/. For each time window, omnibus ANOVAs with factors Group (Signers, Non-Signers), Prime (Related, Unrelated), Laterality (Left, Right, Midline) and Anterior/Posterior (Prefrontal, Frontal, Central, Parietal, Occipital) were conducted, followed by planned follow-up ANOVAs for each group separately. Distributional factors were included to better characterize potential differences in the scalp distributions between groups and across time windows. For this reason, we do not report main effects or interactions including only distributional factors; we only report significant tests that include the primary factors of interest. Partial eta squared (η_p_^2^) is reported as a measure of effect size. Greenhouse-Geisser correction was applied to all effects with more than one degree of freedom in the numerator.

## 3. Results

**Fig. 3.**
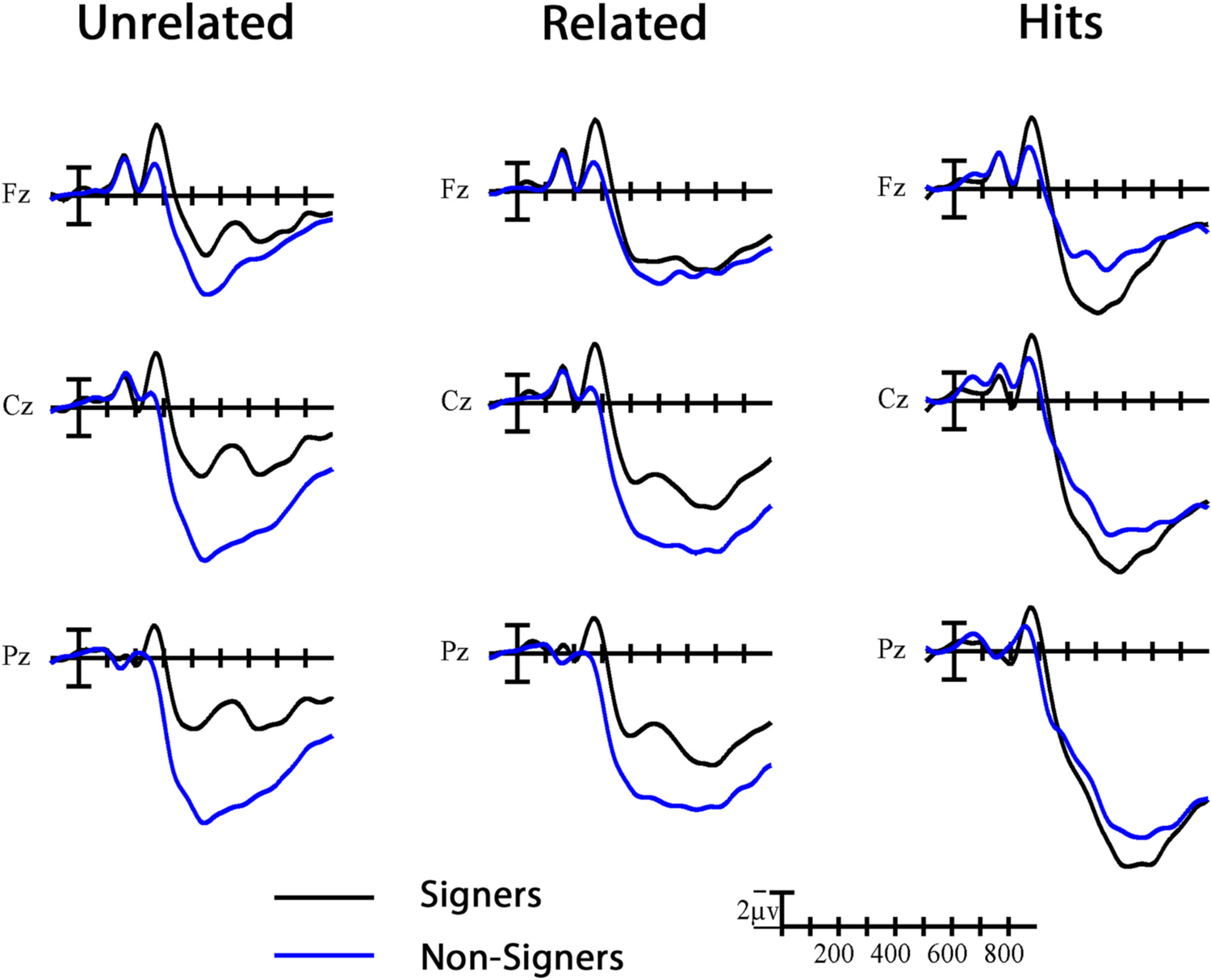
Group effects. Grand average ERP waveforms showing the group effect for all related and unrelated critical trials (collapsed across the three conditions) and hit repetition trials at three representative midline sites. Each vertical tick marks 100 ms and negative is plotted up.

In Fig. 3, the grand average ERPs for each group are plotted at representative midline sites for all critical targets in the unrelated and related conditions (i.e., collapsed across phonological parameters) and hit repetition trials. All significant effects of Group that are reported below reflect larger negativities for the signer group compared to the non-signer group, unless otherwise stated. All significant effects of Prime reflect standard priming effects – larger negativities for the unrelated condition compared to the related condition – unless otherwise stated.

### 3.1. 200–400 Ms

#### 3.1.1. HS + LOC

**Fig. 4.**
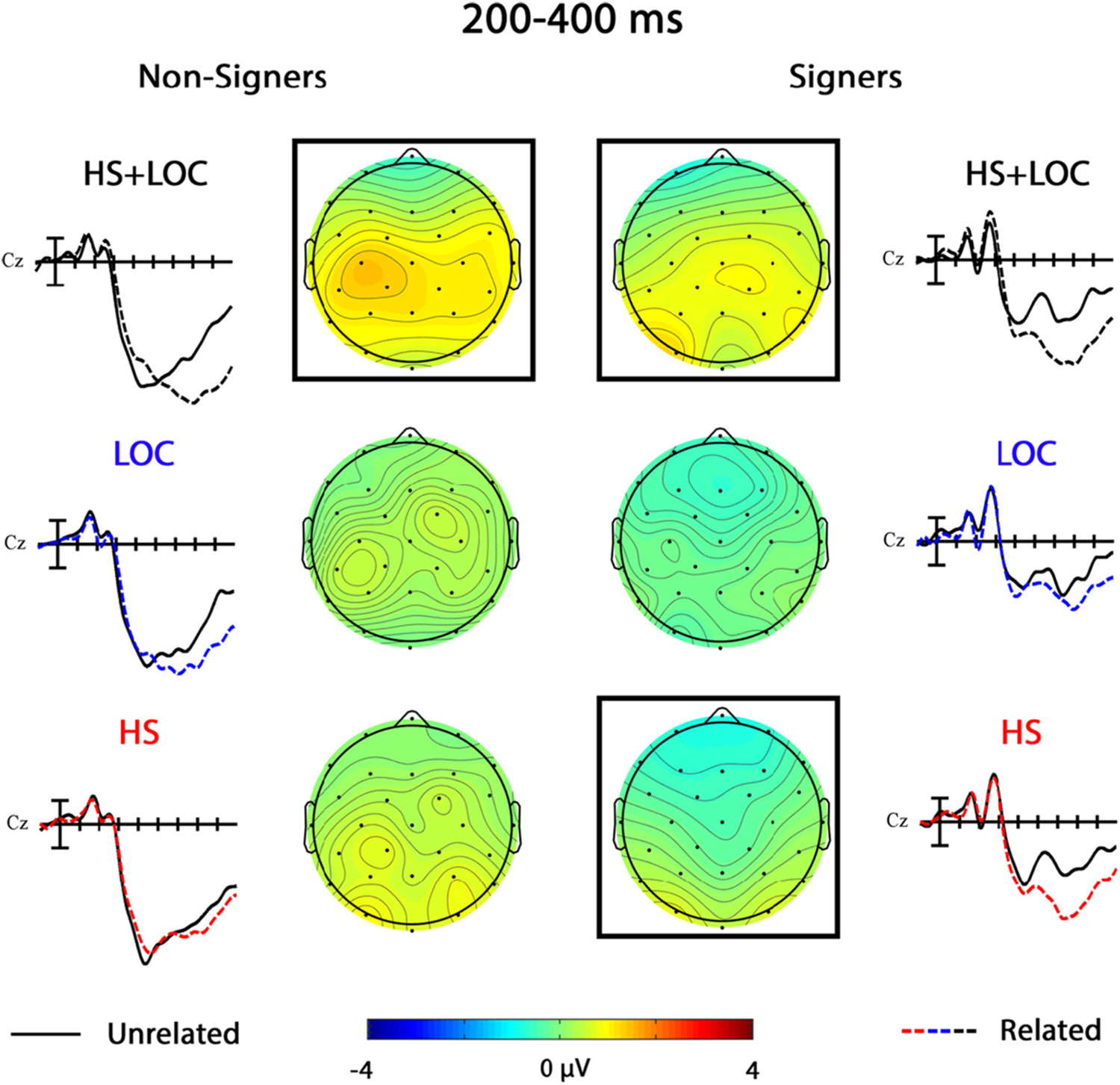
Phonological priming effects 200–400 ms. Grand average ERP waveforms showing the priming effects in the location only (LOC), handshape only (HS), and double parameter (HS + LOC) conditions for the non-signers and signers at representative site Cz. Each vertical tick marks 100 ms and negative is plotted up. Scalp voltage maps show the distributions of the three effects (unrelated-related) for each group. Cool colors represent a priming effect in the standard direction, whereas warm colors represent a “reversed” priming effect. A black box indicates that there was a significant main effect of Prime or a significant interaction involving Prime for that condition and group.

In the omnibus HS + LOC analysis, a significant four-way interaction indicated that the distribution of the N300 priming effect differed between groups, Group × Prime × Laterality × Anterior/Posterior, *F* (8,304) = 2.79, *p* = .029, η_p_^2^ = 0.07 (see Fig. 4). In the non-signer group, targets preceded by HS + LOC related primes elicited the standard priming effect over FP sites, but *larger* negativities for targets in related pairs compared to those in unrelated pairs (i.e., reversed priming) across more central sites, especially on the left side of the scalp, Prime × Anterior/Posterior, *F*(4,76) = 6.28, *p* = .006, η_p_^2^ = 0.25, Prime × Laterality × Anterior/Posterior, *F*(8,152) = 2.50, *p* = .039, η_p_^2^ = 0.12. The pattern in the signer group was similar; the standard priming effect was observed focally across FP electrodes, but the effect reversed over more posterior sites, with the largest difference being at O1, Prime × Laterality × Anterior/Posterior, *F*(8,152) = 5.03, *p* = .005, η_p_^2^ = 0.21.

#### 3.1.2. LOC

In the omnibus LOC analysis, there was a significant main effect of Group, *F*(1,38) = 9.90, *p* = .003, η_p_^2^ = 0.21. There were no significant effects of LOC priming in this window for either the non-signers, all *p*s > 0.63, or the signers, all *p*s > 0.31 (see Fig. 4).

#### 3.1.3. HS

In the omnibus HS analysis, there was a significant main effect of Group, *F*(1,38) = 6.72, *p* = .014, η_p_^2^ = 0.15. There were no significant effects of HS priming for the non-signer group, all *p*s > 0.11. In contrast, in the signer group targets preceded by HS related primes elicited the standard priming effect over the anterior portion of the scalp but *larger* negativities for targets in related pairs compared to those in unrelated pairs (i.e., reversed priming) over the posterior portion of the scalp, Prime × Anterior/Posterior, *F*(4,76) = 4.66, *p* = .018, η_p_^2^ = 20 (see Fig. 4).

### 3.2. 400–600 ms

#### 3.2.1. HS + LOC

**Fig. 5.**
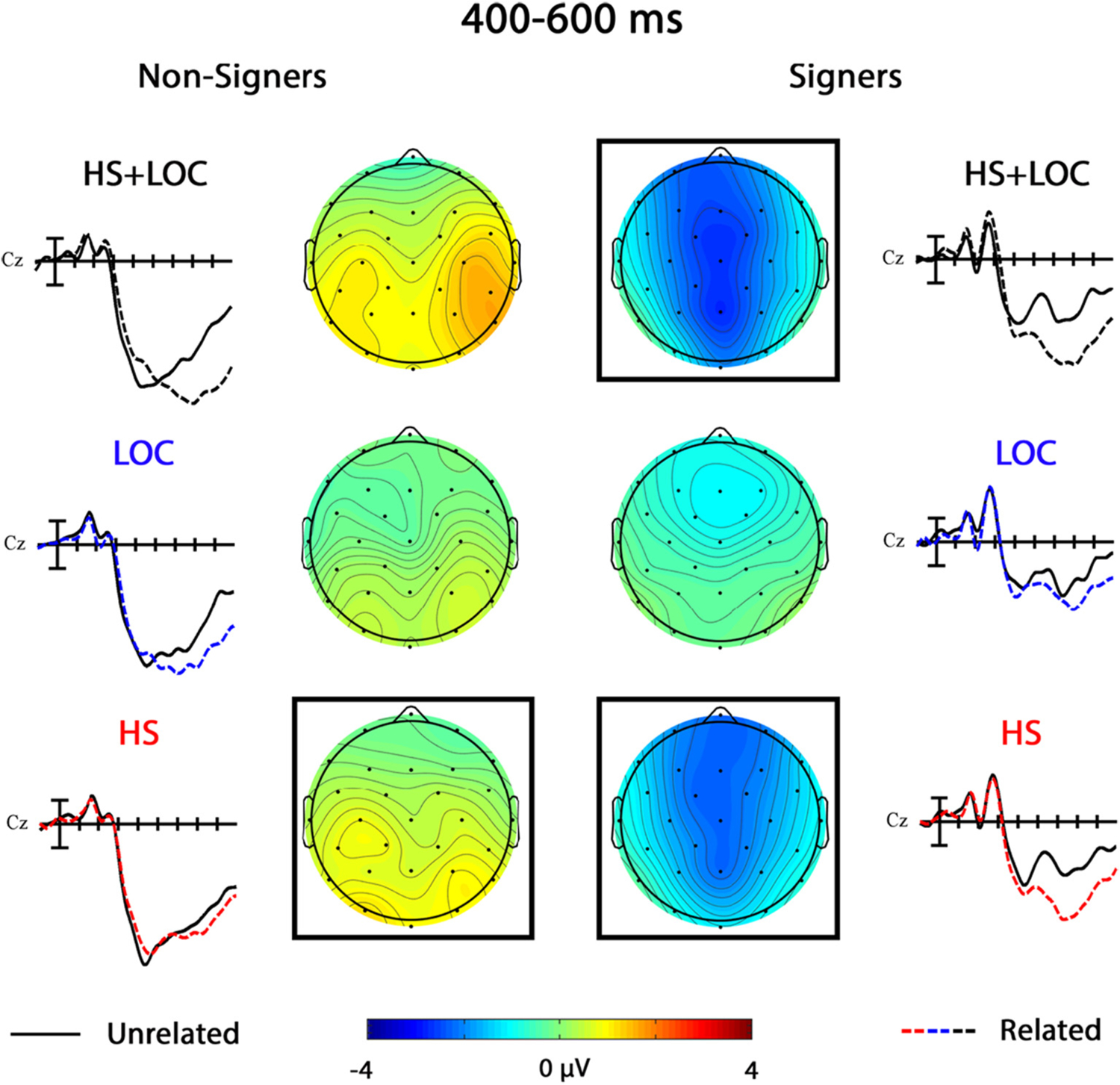
Phonological priming effects 400–600 ms. Grand average ERP waveforms showing the priming effects in the location only (LOC), handshape only (HS), and double parameter (HS + LOC) conditions for the non-signers and signers at representative site Cz. Each vertical tick marks 100 ms and negative is plotted up. Scalp voltage maps show the distributions of the three effects (unrelated-related) for each group. Cool colors represent a priming effect in the standard direction, whereas warm colors represent a “reversed” priming effect. A black box indicates that there was a significant main effect of Prime or a significant interaction involving Prime for that condition and group.

In the omnibus HS + LOC analysis, there was a significant main effect of Group, *F*(1,38) = 6.44, *p* = .015. This difference was especially prominent over centro-parietal electrodes and was slightly reversed (i.e., larger negativity for the non-signers) over the most anterior sites, Group × Anterior/Posterior, *F*(4,152) = 4.85, *p* = .013, η_p_^2^ = 0.11. Moreover, the HS + LOC priming effect differed between groups in this window, Group × Prime, *F*(1,38) = 12.14, *p* = .001, η_p_^2^ = 0.24 (see Fig. 5). There were no significant effects of HS + LOC priming in the non-signer group, all *p*s > 0.07. In contrast, there was a significant main effect of Prime for signers, *F*(1,19) = 13.68, *p* = .002, η_p_^2^ = 0.42, that was strongest at centro-posterior midline sites, Prime × Laterality, *F*(2,38) = 8.36, *p* = .005, η_p_^2^ = 0.30, Prime × Laterality × Anterior/Posterior, *F*(8,152) = 3.00, *p* = .026, η_p_^2^ = 0.14.

#### 3.2.2. LOC

In the omnibus LOC analysis, there was a significant main effect of Group, *F*(1,38) = 13.45, *p* = .001, η_p_^2^ = 0.26, that was especially prominent over midline and centro-posterior sites, Group × Laterality, *F* (2,76) = 4.69, *p* = .014, η_p_^2^ = 0.11, Group × Anterior/Posterior, *F* (4,152) = 4.73, *p* = .016, η_p_^2^ = 0.11. In separate follow-ups, there were no significant effects of LOC priming for the non-signer group, all *p*s > 0.26, or the signer group, all *p*s > 0.17 (see Fig. 5).

#### 3.2.3. HS

There was a main effect of Group in the omnibus HS analysis, *F*(1,38) = 7.23, *p* = .011, η_p_^2^ = 0.16, that was largest across centro-posterior sites, Group × Anterior/Posterior, *F*(4,152) = 5.29, *p* = .010, η_p_^2^ = 0.12. In addition, a significant Group × Prime interaction indicated that the HS priming effect went in opposite directions across groups, *F*(1,38) = 25.40, *p <* .001, η_p_^2^ = 0.40 (see Fig. 5). In the non-signer group, targets preceded by HS related primes elicited *larger* amplitude negativities compared to those preceded by unrelated primes (i.e., reversed priming), especially at occipital sites, Prime × Anterior/Posterior, *F* (4,76) = 4.42, *p* = .018, η_p_^2^ = 0.19. In the signer group, targets preceded by HS related primes elicited *smaller* amplitude negativities than those preceded by unrelated primes (i.e., standard priming), *F*(1,19) = 25.96, *p <* .001, η_p_^2^ = 0.58.

### 3.3. 600–800 ms

#### 3.3.1. HS + LOC

As in the previous window, the effect of Group in the omnibus HS + LOC analysis was strongest at centro-parietal sites, Group × Anterior/Posterior, *F*(4,152) = 5.47, *p* = .007, η_p_^2^ = 0.12. In follow-up analyses by group, there was a significant main effect of Prime for both non-signers, *F*(1,19) = 19.67, *p <* .001, η_p_^2^ = 0.51, and signers, *F*(1,19) = 44.00, *p <* .001, η_p_^2^ = 0.70. The priming effect was strongest over central midline electrodes for non-signers, Prime × Laterality, *F*(2,38) = 11.98, *p <* .001, η_p_^2^ = 0.39, Prime × Laterality × Anterior/Posterior, *F*(8,152) = 3.06, *p* = .020, η_p_^2^ = 0.14, and strongest at midline electrodes for signers, Prime × Laterality, *F*(2,38) = 4.84, *p* = .031, η_p_^2^ = 0.20.

#### 3.3.2. LOC

There was a significant main effect of Group in the omnibus LOC analysis in this later window, *F*(1,38) = 8.00, *p* = .007, η_p_^2^ = 0.17, that was strongest across centro-posterior sites, Group × Anterior/Posterior, *F*(4,152) = 5.91, *p* = .006, η_p_^2^ = 0.13. The main effect of Prime was significant for both non-signers, *F*(1,19) = 11.28, *p* = .003, η_p_^2^ = 0.37, and signers, *F*(1,19) = 5.58, *p* = .029, η_p_^2^ = 0.23. For non-signers, the priming effect was stronger at left hemisphere and midline sites, Prime × Laterality, *F*(2,38) = 3.84, *p* = .034, η_p_^2^ = 0.17.

#### 3.3.3. HS

A Group × Anterior/Posterior interaction in the omnibus HS analysis indicated that mean amplitude in this window was more negative (i.e., less positive) for signers compared to non-signers, especially at centro-posterior sites, whereas the opposite effect was observed at the most anterior sites, *F*(4,152) = 4.48, *p* = .017, η_p_^2^ = 0.10. A Group × Prime interaction further indicated that the HS priming effect was larger for the signer group, *F*(1,38) = 18.89, *p <* .001, η_p_^2^ = 0.33. There were no significant effects of HS priming for non-signers, all *p*s > 0.10. In contrast, there was a significant main effect of Prime for signers, *F*(1,19) = 49.58, *p <* .001, η_p_^2^ = 0.72, that was especially strong across centro-posterior sites, Prime × Anterior/Posterior, *F*(4,76) = 3.74, *p* = .034, η_p_^2^ = 0.16.

### 3.4. Behavior

Although the signer group was numerically faster and more accurate than the non-signer group at identifying hit repetition trials (see Table 1), neither of these effects reached significance, both *p*s > 0.07. In the false alarm omnibus analysis on critical trials, there were also no significant effects involving Group, all *p*s > 0.05 (see Table 2).

In follow-up false alarm analyses conducted separately for each group, there were significant main effects of Prime for both the non-signers, *F*(1,19) = 7.54, *p* = .013, η_p_^2^ = 0.28, and the signers, *F*(1,19) = 7.98, *p* = .011, η_p_^2^ = 0.29. Sign targets preceded by phonologically related primes were more likely to elicit false alarms (i.e., repetition responses) overall. Significant main effects of Parameter for the non-signers, *F*(2,38) = 6.16, *p* = .012, η_p_^2^ = 0.24, and signers, *F*(2,38) = 7.43, *p* = .007, η_p_^2^ = 0.28, further indicated that the false alarm rate differed for targets across the various conditions. Significant Prime × Parameter interactions for the non-signers, *F*(2,38) = 12.43, *p <* .001, η_p_^2^ = 0.40, and signers, *F*(2,38) = 6.54, *p* = .014, η_p_^2^ = 0.26, indicated that the effect of prime relatedness differed for the various parameters. In both groups, the effect of prime relatedness on false alarm rates was significantly larger in the HS + LOC condition than in either of the other two conditions, all *p*s < 0.036. In non-signers, the effect of prime relatedness on false alarm rates did not significantly differ between the HS and LOC conditions, *F*(1,19) = 3.11, *p* = .094, η_p_^2^ = 0.14. In signers, the effect of prime relatedness on false alarm rates significantly differed between these two conditions, *F*(1,19) = 5.44, *p* = .031, η_p_^2^ = 0.22. It went in the expected direction (i.e., more false alarms when targets were preceded by related primes compared to unrelated primes) for HS and in the opposite direction for LOC.

## 4. Discussion

To better understand the mechanisms that underlie phonological priming, we used ERPs to compare the effects of form priming in a repetition detection task between deaf signers and hearing non-signers. Signers have established sublexical and lexical representations of ASL phonological structure. Non-signers can perceive form-based similarity in ASL, but do not have any associated linguistic representations. Signed languages afford a unique opportunity to address this issue; analogous analyses are not possible in spoken languages, as there is no control group that can perceive speech but has not acquired a sound-based phonological system. Targets in critical trials were unrelated to the preceding prime or were phonologically related in one of three ways: overlap in handshape only, location only, or both handshape and location. The goal was to determine whether perceptual similarity contributes to phonological priming, in which case we expected an early priming effect that was similar across groups. Our findings suggest that non-signers were sensitive to the increased perceptual similarity between phonologically related prime and target signs. They had similar accuracy and reaction times as the signers when identifying full repetitions, and both groups were most likely to erroneously press for targets in the related HS + LOC condition. There was also some evidence of an early reversed N300 priming effect that was similar across groups and most prominent for the HS + LOC condition. However, ERP priming effects subsequently diverged as a function of both linguistic knowledge and the specific parameters that were manipulated. Thus, although perceptual similarity may contribute to phonological priming, the effects are primarily driven by pre-activation of linguistic representations.

The N300 window was dominated by a reversed priming effect that was observed for both groups in the HS + LOC condition, albeit with slightly different scalp distributions. We suggest that these early reversed priming effects reflect perceptual similarity. Similarities between groups prevailed in the two-parameter comparison, when perceptual similarity was maximized. However, the single parameter conditions offered a more nuanced picture of how perceptual similarity was affected by differential ASL knowledge in the two groups. The posterior reversed-priming effect in the handshape-only condition emerged during the N300 for signers, but not until the N400 window for non-signers. In contrast, location overlap by itself did not seem to elicit this reversed N300 priming effect at all in either group. Thus, a lifetime of ASL knowledge and experience appear to tune perceptual processes such that signers are more attuned than non-signers to handshape information. This conclusion is consistent with the finding that signers (in contrast to non-signers) exhibit categorical perception effects for handshape, i.e., better discrimination across than within handshape categories (Baker, Idsardi, Golinkoff, & Petitto, 2005; Emmorey et al., 2003; Palmer et al., 2012).

A potential generator for these early perceptual similarity effects is the extrastriate body area that has been shown to selectively respond to human hands and arms (e.g., Schwarzlose, Baker, & Kanwisher, 2005; Taylor, Wiggett, & Downing, 2007). We speculate that repetition expectations for body postures (HS + LOC condition) and hand configurations (HS condition) may have engendered a repetition enhancement effect, resulting in greater neural activity in this early time window for target signs in the related conditions relative to the unrelated conditions. Repetition enhancement in extrastriate visual cortex is argued to be associated with prediction signals, which are boosted by repetition (e.g., de Gardelle, Waszczuk, Egner, & Summerfield, 2013). The association between these effects and the early reversed priming effect that we found here remains tentative and warrants further research; the anatomical underpinnings of phonological priming effects in signed languages remain poorly understood.

Signers’ early sensitivity to handshape overlap carried over into the N400 window. Specifically, the N400 window in the signer group was dominated by sizeable priming effects in the standard direction that were nearly identical for targets in the HS + LOC and HS conditions, but not the LOC condition (see Fig. 7). These results mirror previous studies and emphasize the need for a consideration of the role that each parameter plays in sign recognition (e.g., Gutiérrez et al., 2012). Although null effects must be interpreted with caution, it is plausible that the absence of a location priming effect in the signer group was due to the combined influence of sublexical facilitation and interference from lexical competition (see Gutiérrez et al., 2012; Meade et al., 2021, for further discussion). In contrast, we attribute the lack of N400 priming effects across all conditions in the non-signer group to the absence of linguistic representations. Neither the primes nor the targets could be processed linguistically, and therefore their shared sublexical characteristics and their proximity in the lexical network had no effect on processing in the non-signers.

Further evidence that signers were treating the signs as meaningful linguistic symbols in a way that was inaccessible to non-signers comes from the overall effects of Group. Across the three comparisons that included different target items, N400 amplitude was larger for signers than for non-signers (refer to Fig. 3). Building on previous research using the Stroop task (e.g., Bosworth, Binder, Tyler, & Morford, 2021; Dupuis & Berent, 2015), this finding confirms that the sign stimuli elicited automatic lexico-semantic processing in the signers despite the relatively shallow task. That these visual-manual stimuli would elicit larger N400s in the signers was not a foregone conclusion; several studies have found that manual gestures elicit componentry in hearing non-signers that resembles that of sign processing, including a prominent negative peak in the N400 window (e.g., Özyürek, Willems, Kita, & Hagoort, 2007; Wu & Coulson, 2005, 2007). The difference between those studies and the present study is that the gestures that are typically used are iconic and transparent. That is, the gestures can still be processed for meaning by non-signers. In a post-hoc analysis, we calculated the mean iconicity ratings from hearing non-signers for the 134 (out of 138) target signs in the present study from ASL-LEX (http://asl-lex.org; Sehyr, Caselli, Cohen-Goldberg, & Emmorey, 2021). On a scale from 1 (not iconic at all) to 7 (very iconic), the average iconicity rating was 2.97 (SD 1.58). The relatively low iconicity of the target signs together with a task that did not encourage meaning processing or guessing could explain the absence of an N400 in the non-signers.

**Fig. 6.**
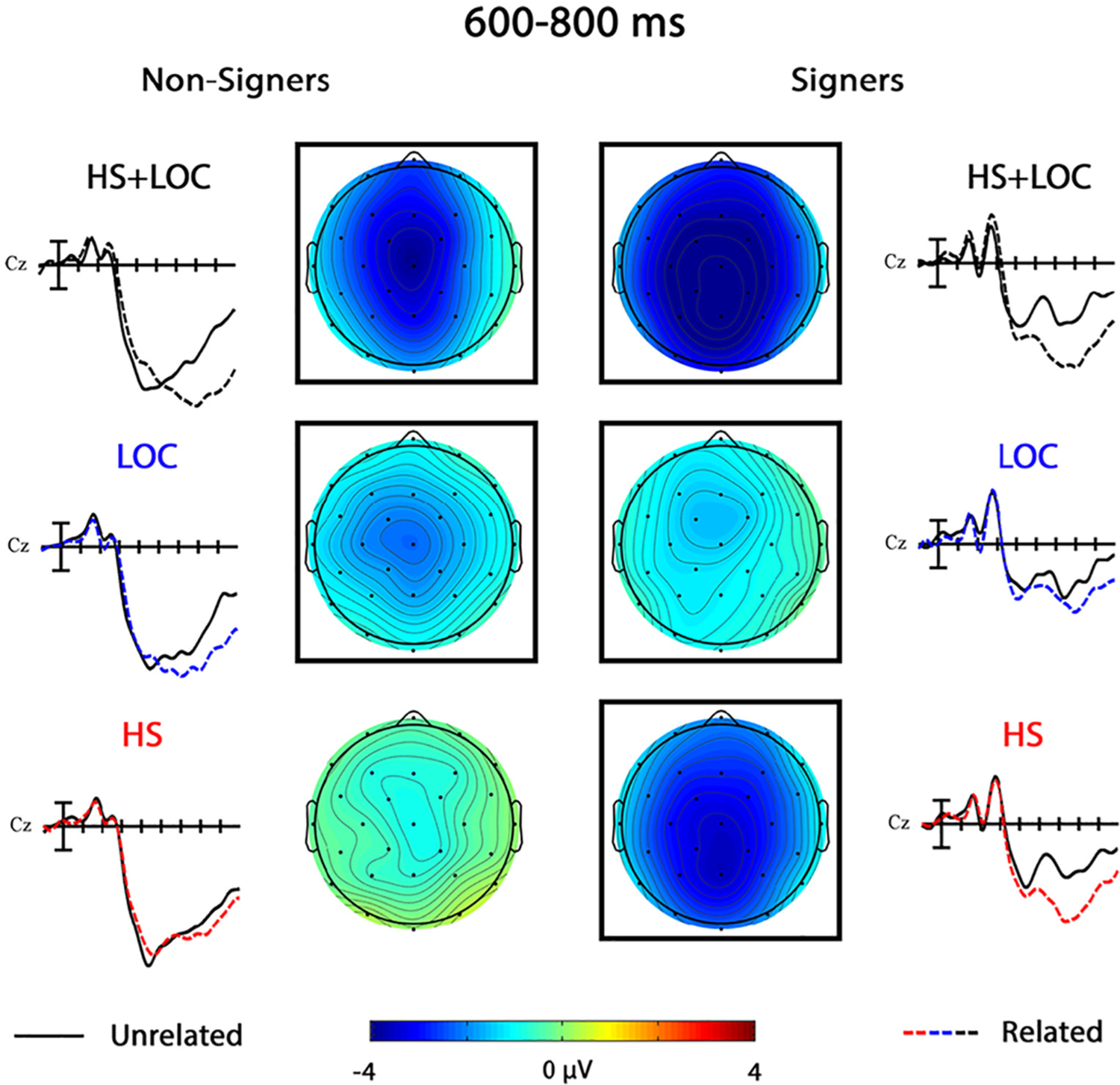
Phonological priming effects 600–800 ms. Grand average ERP waveforms showing the priming effects in the location only (LOC), handshape only (HS), and double parameter (HS + LOC) conditions for the non-signers and signers at representative site Cz. Each vertical tick marks 100 ms and negative is plotted up. Scalp voltage maps show the distributions of the three effects (unrelated-related) for each group. Cool colors represent a priming effect in the standard direction, whereas warm colors represent a “reversed” priming effect. A black box indicates that there was a significant main effect of Prime or a significant interaction involving Prime for that condition and group.

**Fig. 7.**
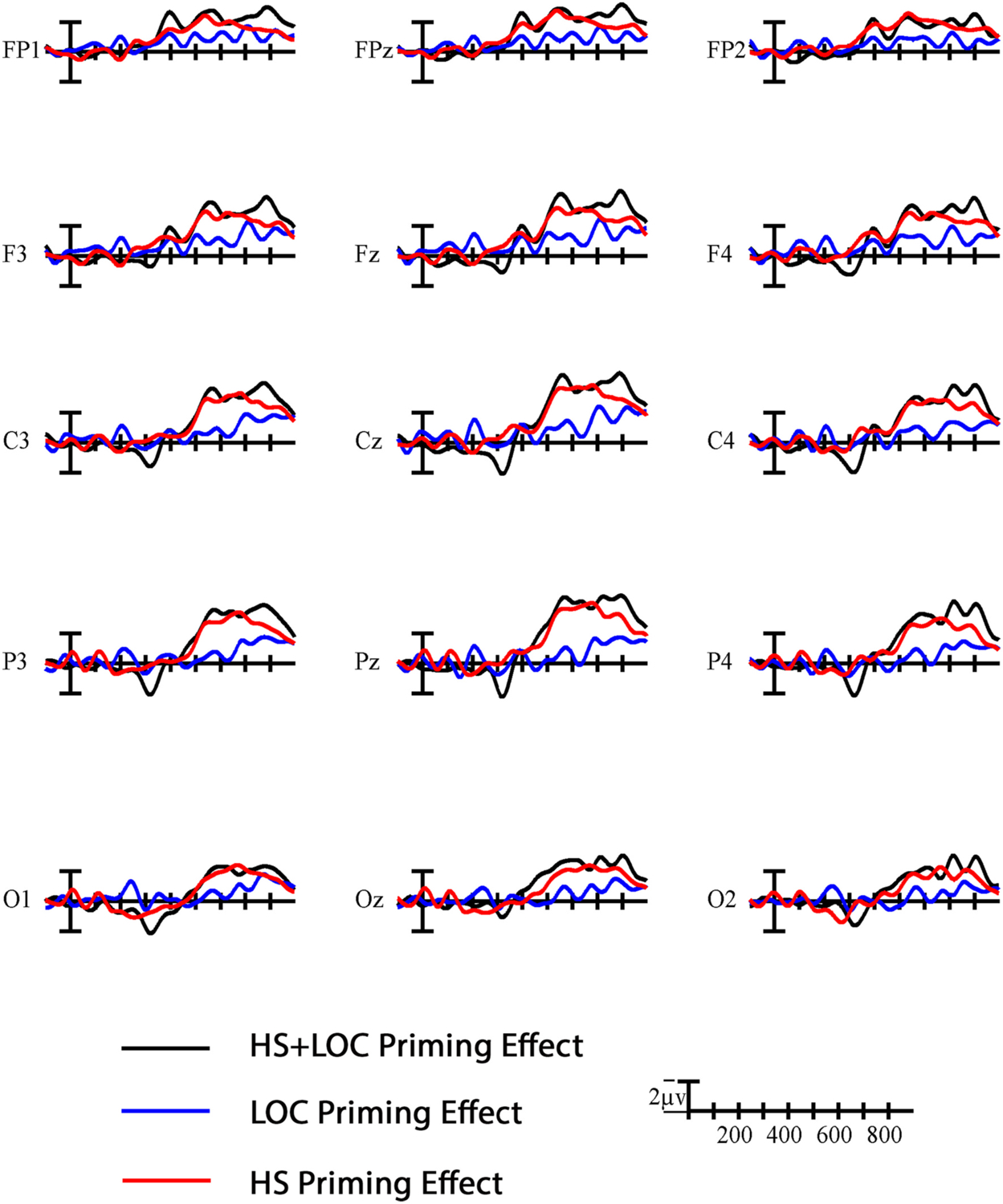
Difference waves for signers. Difference waves showing the effect of phonological relatedness (unrelated-related) across the three conditions in the signer group. Each vertical tick marks 100 ms and negative (i.e., a standard priming effect) is plotted up. The calibration bar marks 2 μV.

**Fig. 8.**
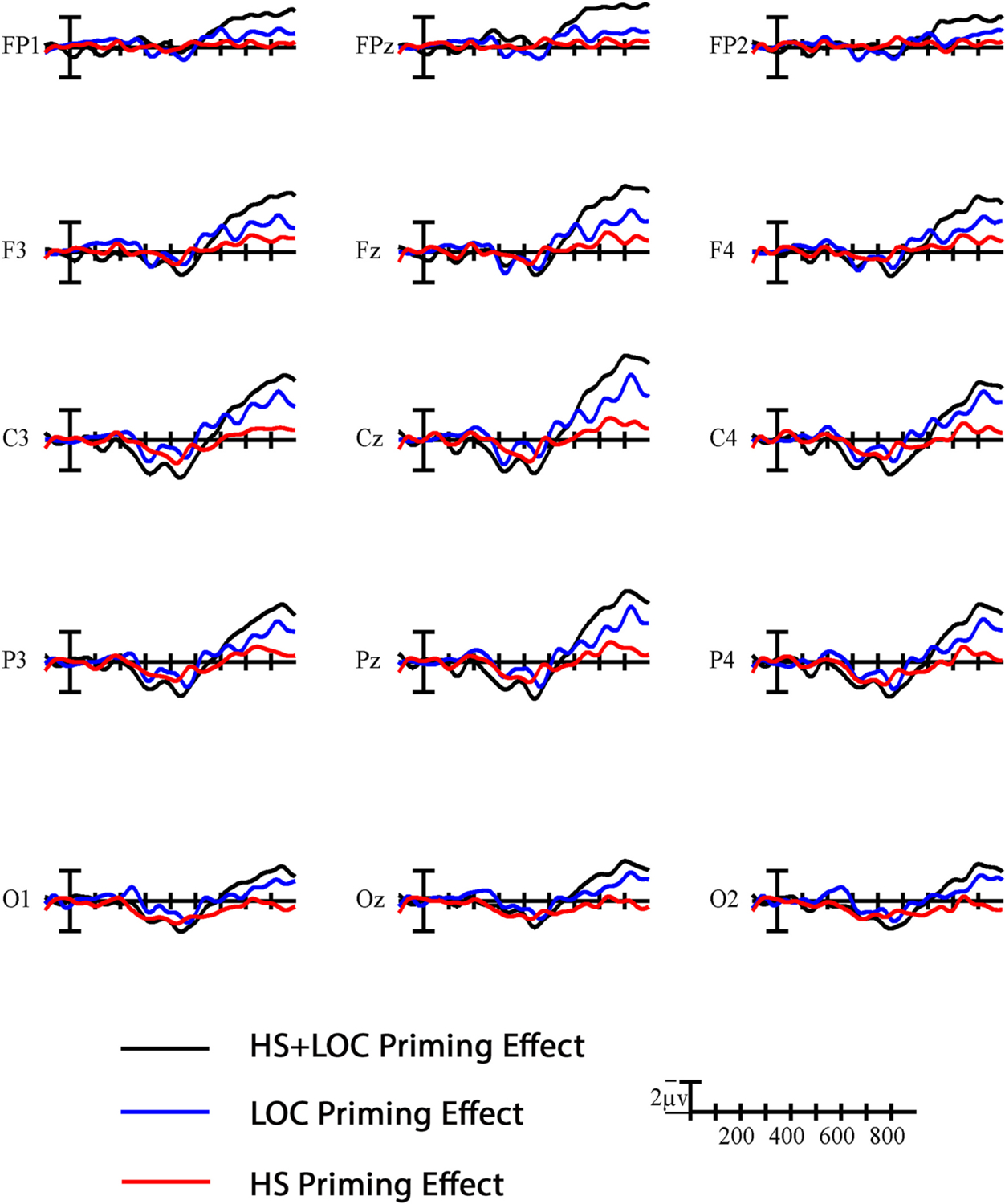
Difference waves for non-signers. Difference waves showing the effect of phonological relatedness (unrelated-related) across the three conditions in the non-signer group. Each vertical tick marks 100 ms and negative (i.e., a standard priming effect) is plotted up. The calibration bar marks 2 μV.

In the late window and behavioral responses, the nuances of how each group was processing these signs and making their decision came once again from the conditions in which only one parameter was manipulated. As in the N400 window, the handshape priming effect patterned with the two-parameter condition in signers (see Fig. 7). Targets in the related handshape condition also elicited more false alarms than those in the location condition, in the signer group only. These patterns further suggest that signers were relying on handshape similarity to inform their decisions. In contrast, handshape priming was not significant in this window for the non-signers, who appeared to be relying more on location information to make their decisions. Indeed, these late effects of location and two-parameter priming in the non-signers are the only time that we found significant priming effects in the standard direction (see Figs. 6 and 8). These results extend previous perceptual similarity rating studies (e.g., Hildebrandt & Corina, 2002) to suggest that non-signers’ on-line processing of signs is also largely dominated by the saliency of the location parameter.

The overall effects of group, characterized as larger amplitude positivities for the non-signer group compared to the signer group (see Fig. 3), persisted into this late window. All targets appear to have elicited this large positivity in the non-signer group, even targets in unrelated conditions that shared *no* parameters with the preceding prime. This pattern indicates that these pairs were still being considered as potential repetitions into this late time window. In contrast, signers appear to have had a more efficient “filter” that limited their consideration of potential repetitions to targets that shared handshape (with or without location) with the preceding prime. Their experience with a visual-manual phonology and the linguistic representations that they have developed as a result contribute to this increase in efficiency with regard to sign processing.

Taken together, this comparison of form priming effects between non-signers and signers has demonstrated that the N400 phonological priming effects that have previously been reported are linguistic in nature, due to interactions among sublexical and lexical representations. The non-signers were sensitive to some extent to the perceptual similarity between primes and targets, and this similarity impacted their decisions in the repetition detection task. However, knowledge of the linguistic system influenced the type of information to which participants were attuned. Whereas non-signers considered all targets as potential repetitions, signers efficiently extracted handshape information and used that information to narrow their consideration of potential repetitions. In other words, we have demonstrated that the N400 is sensitive to the interplay between perceptual processing and linguistic experience. Although this conclusion presumably extends to all linguistic systems, irrespective of modality, we were able to capitalize on the visual-manual phonological system of ASL to provide uniquely convincing empirical evidence to this effect.

## Figures and Tables

**Table 1:** Accuracy and reaction times for hit trials [mean (SD)].

	Accuracy	RTs
Non-signers	41.6 (3.5)	1003 ms (330 ms)
Signers	43.2 (3.4)	853 ms (146 ms)

Note: There were a total of 46 repetition probes.

**Table 2:** False alarm rates for critical trials per condition [mean (SD)].

	HS + LOC		LOC		HS	
	Related	Unrelated	Related	Unrelated	Related	Unrelated
Non-signers	1.7 (2.1)	0.0 (0.0)	0.5 (0.9)	0.2 (0.4)	0.3 (0.6)	0.5 (0.8)
Signers	1.1 (1.5)	0.2 (0.4)	0.0 (0.0)	0.2 (0.4)	0.4 (0.5)	0.2 (0.4)

Note: There were 46 trials in each of the six critical conditions.
